# The East Asian Erotic Picture Dataset and Gender Differences in Response to Opposite-Sex Erotic Stimuli in Chinese College Students

**DOI:** 10.3389/fpsyg.2021.648271

**Published:** 2021-04-21

**Authors:** Qianqian Cui, Zixiang Wang, Ziyuan Zhang, Yansong Li

**Affiliations:** ^1^Reward, Competition and Social Neuroscience Lab, Department of Psychology, School of Social and Behavioral Sciences, Nanjing University, Nanjing, China; ^2^Department of Applied Foreign Language Studies, Nanjing University, Nanjing, China; ^3^Institute for Brain Sciences, Nanjing University, Nanjing, China

**Keywords:** erotic stimuli, heterosexual, gender differences, East Asia, sex

## Abstract

Understanding the processing of sexual stimuli has become a significant part of research on human sexuality. In addition to individual characteristics (gender and sexual orientation), empirical studies have shown that cultural factors play an important role in sexual stimuli processing. The attitudes toward sex have been reported to be more conservative in East Asian societies as compared to western countries, and significantly more sexual difficulties are observed among East Asian people. However, stimulus materials, which potentially facilitate human sexuality research on native East Asian people, are relatively not satisfactory. Erotic stimuli depicting East Asian figures are limited in the existing picture datasets. To address this issue, we present a collection of 237 erotic and 108 control pictures, accompanied by self-reported ratings of sexual arousal, pleasantness, and sexual attractiveness for opposite-sex erotic stimuli by heterosexual males and females (*n* = 40, divided into two equal-sized subsamples). This collection is divided into six categories, depending on their contents: dressed males (44), semi-nude males (65), nude males (64), dressed females (64), semi-nude females (52), and nude females (56). We showed gender differences in sexual arousal, pleasantness, and sexual attractiveness ratings in response to opposite-sex erotic pictures. Males reported the highest levels of sexual arousal, pleasantness, and sexual attractiveness for nude female pictures, whereas females reported the highest levels of sexual arousal, pleasantness, and sexual attractiveness for semi-nude male pictures. The erotic picture dataset may provide a useful resource of erotic stimuli that can be used as stimulus materials in experimental research on sexual function in East Asians.

## Introduction

Human sexual behavior is a means of reproduction and survival strategy (Hamilton, 1964), which is motivated by sexual pleasure (Tidon and Lewontin, 2004). Erotic pictures displaying real humans have been used to facilitate the research on human sexual behavior. Both facial and bodily cues reliably indicate gender, health, and fertility. Waist-to-hip ratio (WHR) is recognized to be a signal of health in both genders (Pouliot et al., 1994) and a signal of fertility in females (Bowman and Singh, 1993). Facial features (i.e., symmetry and sexual dimorphism) are also potential indicators of health (Valko et al., 2006). Humans and other primates have been observed to show highly selective preferences for viewing the sexually relevant signals of conspecifics (Grammer et al., 2003; Deaner et al., 2005). Physiological arousal responses are supposed to follow when the sexually relevant contents are perceived to be positive. This process is considered to subsequently facilitate sexual behaviors and ultimately copulation (Walen and Roth, 1987).

A variety of methodologies have been adopted in empirical studies to investigate human sexual responses when exposed to visual erotic stimuli. Classic approaches include subjective self-report and genital response measurements on sexual arousal (Chivers et al., 2004; Rupp and Wallen, 2009), as well as neuroimaging research (Safron et al., 2007). Empirical literature has emphasized that sexual responses toward erotic stimuli are subject to individual differences (Georgiadis and Kringelbach, 2012), gender differences (Rupp and Wallen, 2008, 2009; Stoléru et al., 2012; Sylva et al., 2013), and culture differences (Yule et al., 2010). Males generally tend to report increased subjective sexual arousal when being exposed to specific categories of erotic stimuli such as nude female figures. Females do not display similar category-specific preferences when viewing a variety of erotic stimuli. Genital measurement in the presence of same and opposite-sex erotic stimuli displays the similar pattern: males showed the highest genital arousal when responding to their preferred sex, whereas female displayed comparable genital arousal irrespective of the sex of the actors (Chivers et al., 2004). Males were also reported to give higher ratings than females when asked to indicate how attractive and sexually arousing they find the visual erotic stimuli are (Money and Ehrhardt, 1972; Laan et al., 1994).

In addition to gender differences, empirical studies have revealed that sexual attitudes and difficulties are also significantly associated with culture and ethnicity. It is theorized that the strict moral standards and social expectations in modern Asian culture contribute to the suppression of sexual needs and expression, while the contemporary western culture is considered to have relatively more liberal attitude toward sexual expression and behavior (Yule et al., 2010). When compared to Caucasian-Canadian college students, Asian-Canadian college students reported significantly more conservative attitudes toward all measures of interpersonal behavior (i.e., sexual intercourse) and sociosexual restrictiveness (i.e., lifetime number of sexual partners) across gender (Meston et al., 1996). In another study on acculturation and sexual function among Canadian women, significantly more anxiety from anticipated sexual activity was reported in Asian-Canadian women than their Euro-Canadian counterparts (Yule et al., 2010). A global study of sexual attitudes and behaviors, which included 14,000 women in 29 different countries, also reported that lack of sexual interest, inability to reach orgasm, finding sex not pleasurable, pain during sex, and lubrication difficulties were higher in East Asian areas than in Europe and North America (Nicolosi et al., 2005).

Although empirical studies have proved the impact of eastern-western culture differences on human sexuality, the majority of the participants in previous studies were Euro/North American residents with East Asian Ethnicity. Native East Asian residents have not yet received extensive attention. Despite the large population in China, research regarding sexual responses with a specific focus on Chinese males and females are rather inadequate. Furthermore, the visual erotic stimuli used in past human sexuality-related experiments are not satisfactory to future research on native East Asian participants. The International Affective Picture System (IAPS), which is one of the most common recognized databases of standardized visual affective stimuli, offers a limited set of sexual stimuli in addition to a variety of images from other categories (Lang et al., 2008). Yet those sexual stimuli are argued to be outdated for experimental research (Jacob et al., 2011). Recently, increasing efforts were devoted to complementing the IAPS dataset. Jacob et al. (2011) introduced a set of 100 erotic pictures including two categories – intimate heterosexual couples and attractive single males. All images are not sexually explicit (i.e., no genitals are included). In addition, Rupp and Wallen (2009) created a set of 216 sexually explicit photographs of heterosexual couples in the process of intercourse. An erotic subset for the Nencki Affective Picture System (NAPS) featuring categories of individual males, individual females, opposite-sex couples, and same-sex couples has also been developed to facilitate future research on both gay men and lesbian women (Wierzba et al., 2015).

However, as outlined above, the existing erotic picture databases are constituted by dominantly erotic stimuli depicting Caucasian figures. The lack of East Asian picture stimuli leaves open the possibility that East Asian participants may perceive Caucasian stimuli differently presenting a serious confounding especially in any study comparing Caucasian and East Asian participants’ responses to such stimuli. Previous research has documented that individuals’ discriminatory attitudes in relation to race/ethnicity are formed through throughout adolescence (Benner and Graham, 2013), which has longstanding effects on individual differences in race/ethnicity-based preferences in adulthood. These preferences may extent to the sexual realm. It has also been found that adults are prone to stereotypes regarding outgroups (Cuddy et al., 2009), and race/ethnicity-based ratings of sexual arousal to erotic stimuli have been reported among racially or ethnically diverse groups (Reed Hughes and Anderson, 2007). It is therefore important to be able to present East Asian participants with stimuli that correspond with their ethnic group. On the other hand, no other picture databases particularly depicting East Asian characters have been offered or validated. Therefore, introducing an erotic picture dataset that depict East Asian figures and investigating the sexual responses of native East Asian participants could have practical implications for future sexuality research on culture differences, and also help us to gain a better understanding of determinants of sexual response patterns in general. The erotic image dataset included in this paper is designed to address some of the limitations mentioned above. In the process of selecting visual erotic stimuli, we are primarily interested in pictures derived from non-professional collections, depicting multiple levels of explicitness of sexual contents in a natural manner. Width, height, luminance, contrast, and color hue are adjusted across the collection. The following issues will be covered: (1) present an overview of the East Asian erotic picture dataset: subjective ratings of opposite-sex erotic pictures given by native East Asian participants with respect to multiple rating dimensions are provided and (2) explore an influence of gender on the preferred erotic stimuli. We hypothesized that the ratings would display significant differences with respect to erotic stimuli categories. When viewing opposite-sex pictures, native Chinese males and females would display different category-specific preferences for visual erotic stimuli.

## Materials and Methods

### Participants

A total of 40 heterosexual college students (20 males and 20 females) with a mean age of 22.35 years from Nanjing University participated in the experiment. Those who are under 18 years old were excluded from the experiment. All participants were exclusively heterosexual (Kinsey 0) based on the screening process with the Kinsey Scale (Kinsey et al., 1998). Given that anxiety and depression have been found to affect individuals’ sexual performance (Laurent and Simons, 2009; Rajkumar and Kumaran, 2015), the Beck Depression Inventory (BDI; Beck et al., 1961) and the Zung Self-Rating Anxiety Scale (SAS; Zung, 1971) were employed in order to control for their possible influence. The BDI scores of all participants ranged from 0 to 11, with a mean of 6.10 and a standard deviation of 2.962. The SAS scores of all participants ranged from 21 to 45, with a mean of 25.63 and a standard deviation of 9.059. There is no significant differences in BDI and SAS scores between male and female participants (BDI: *t*(38) = 0.309, *p* > 0.05; SAS: *t*(38) = 0.656, *p* > 0.05). Two participants did not finish the task and their data were excluded from data analysis. All participants have given informed consent to participate. This study was approved by the Nanjing University Institutional Review Board.

### Erotic Stimuli

**Figure 1:**
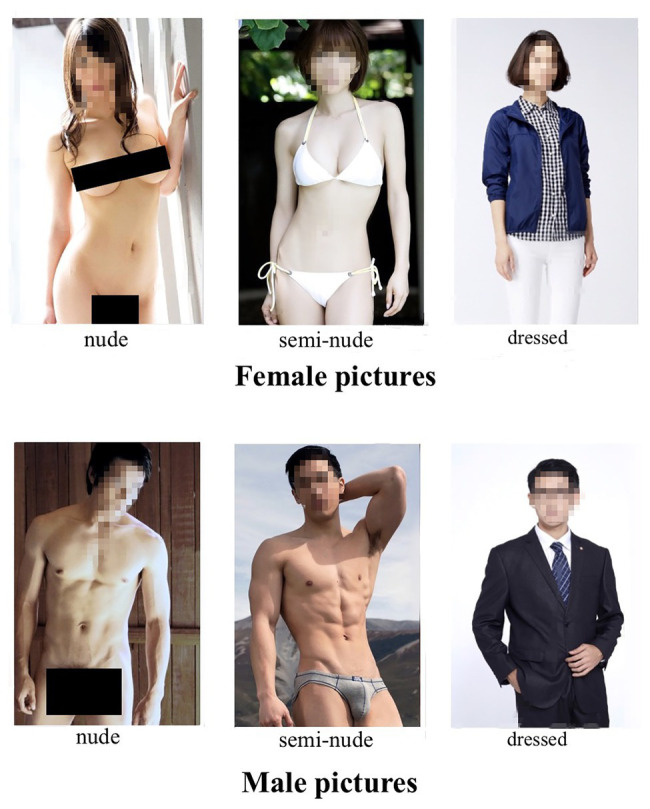
A sample image of males and females from each category.

The erotic stimuli were 345 digital photographs of frontal poses of normal-weight and attractive East Asian adult males and females. Based on the levels of sexual explicitness, the pictures are divided into six categories: (1) dressed male pictures (44), (2) semi-nude male pictures (65), (3) nude male pictures (64), (4) dressed female pictures (64), (5) semi-nude female pictures (52), and (6) nude female pictures (56). The control pictures (dressed ones) were acquired from non-royalty websites and the semi-nude and nude pictures were obtained from pornographic websites, which permits non-commercial use. Logos or emblems were removed and pictures showing piercing or explicit emotional facial expressions (laughing, frowning, etc.) were excluded. All models in the non-erotic category are dressed in sexually non-revealing clothing (sleeved shirt or outwear and long pants/skirts). Each male model in the semi-nude category is dressed in underwear or shorts and displays chest (genital region is covered). Similarly, each female model in the semi-nude category wears bikini or a bra and panty set (chest and the genital region are covered). All models in the nude category clearly display chests and genital regions. The amount of pubic hair was modest and penis and breast size vary modestly across nude category. All the stimuli were adjusted into 500 × 800 pixels. Figure 1 shows examples of males and females from each category. Pictures and ratings are available on reasonable request from the corresponding author (you may also download them on this website: https://yansonglilab.github.io).

### Procedure

The stimuli were, with E-prime 2.0 software (Psychology Software Tools, Pittsburgh, PA, United States) displayed on a 14-inch monitor (120 Hz refresh rate) with a 1920 × 1,080 resolution laptop. Participants were asked to give self-paced ratings of all the opposite-sex pictures based on three dimensions – sexual attractiveness (ranging from “sexually unattractive” to “sexually attractive”), pleasantness (ranging from “not at all pleasant” to “pleasant”), and sexual arousal (ranging from “calm” to “very excited”). In each trial, a single picture was presented to participants in the center of the computer screen and participants were required to report sexual arousal, pleasantness or sexual attractiveness ratings on seven-point scales successively. These three self-reported ratings were counterbalanced across participants. There were 173 trials for females and 172 trials for males, with a scheduled short break every 60 trials. Before the normal experiment, all participants firstly completed a practice program to get familiar with the experimental task.

### Data Analysis

Regarding females’ ratings for male pictures (sexual arousal, pleasantness, and sexual attractiveness), three separate one-way ANOVAs with category (dressed vs. semi-nude vs. nude) as a within-participant factor were performed. In contrast, with regard to males’ ratings for female pictures, due to a violation of homogeneity of variance, three separate Welch’s heteroscedastic F tests and a Games-Howell *post-hoc* test.

## Results

### The Females’ Ratings for Male Pictures

#### Sexual Arousal

**Figure 2:**
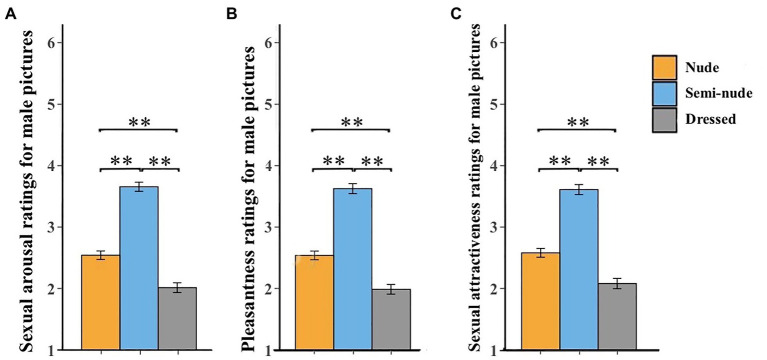
Females’ ratings of sexual arousal **(A)**, pleasantness **(B)**, and sexual attractiveness **(C)** for male pictures. Error bars represent standard errors of the mean. ^**^p < 0.01.

A significant effect of category on females’ sexual arousal ratings for male pictures was found [*F*(2, 170) = 122.69, *p* < 0.001, $\eta_{p}^{2}$ = 0.59; Figure 2A]. The *post-hoc* analysis revealed that females’ sexual arousal ratings for semi-nude male pictures (*M* = 3.66, *SE* = 0.07) were significantly higher than those for both nude male pictures (*M* = 2.54, *SE* = 0.07, *p* < 0.001) and dressed ones (normally clothed; *M* = 2.01, *SE* = 0.08, *p* < 0.001; Table 1). Moreover, the sexual arousal ratings for nude male pictures were significantly higher than those for dressed ones (*p* < 0.001). These results suggest that females rated semi-nude male pictures as more sexual arousing than the other two types of male pictures.

**Table 1:** Females’ sexual arousal, pleasantness, and sexual attractiveness ratings for male pictures.

Ratings	Male pictures		
	Nude	Semi-nude	Dressed
	(Mean ± SE)	(Mean ± SE)	(Mean ± SE)
Sexual arousal	2.54 ± 0.04	3.66 ± 0.05	2.01 ± 0.06
Pleasantness	2.59 ± 0.04	3.62 ± 0.05	2.09 ± 0.07
Sexual attractiveness	2.54 ± 0.04	3.63 ± 0.05	1.97 ± 0.07

#### Pleasantness

Similarly, there was also a significant effect of category on females’ pleasantness ratings for male pictures [*F*(2, 170) = 94.49, *p* < 0.001, $\eta_{p}^{2}$ = 0.53; Figure 2B]. The *post-hoc* analysis revealed that females’ pleasantness ratings for semi-nude male pictures (*M* = 3.62, *SE* = 0.08) were significantly higher than those for both nude male pictures (*M* = 2.59, *SE* = 0.07, *p* < 0.001) and dressed ones (normally clothed; *M* = 2.09, *SE* = 0.08, *p* < 0.001; Table 1). Furthermore, their pleasantness ratings for nude male pictures were significantly higher than those for dressed ones (*p* < 0.001). These findings indicated that females rated semi-nude male pictures as more pleasant than the other two types of male pictures.

#### Sexual Attractiveness

Finally, a significant effect of category on females’ sexual attractiveness ratings for male pictures was also found [*F*(2, 170) = 111.38, *p* < 0.001, $\eta_{p}^{2}$ = 0.57; Figure 2C]. The *post-hoc* analysis revealed that females’ sexual attractiveness ratings for semi-nude male pictures (*M* = 3.63, *SE* = 0.08) were significantly higher than those for both nude male pictures (*M* = 2.54, *SE* = 0.07, *p* < 0.001) and dressed ones (normally clothed; *M* = 1.99, *SE* = 0.08, *p* < 0.001; Table 1). Additionally, females’ sexual attractiveness ratings for nude male pictures were significantly higher than those for dressed ones (*p* < 0.001). These results demonstrated that females rated semi-nude male pictures as more sexual attractiveness than the other two types of male pictures.

### The Males’ Ratings for Female Pictures

#### Sexual Arousal

**Figure 3:**
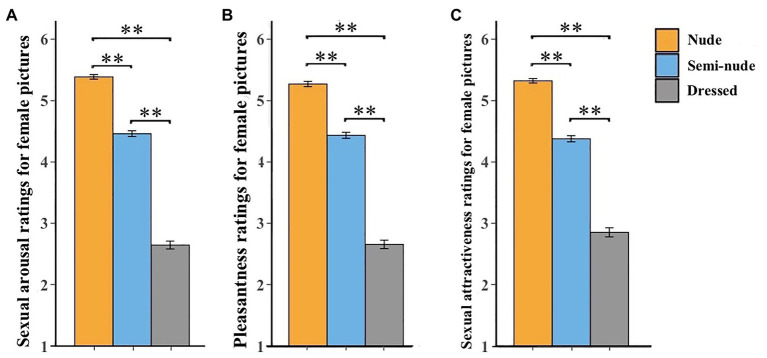
Males’ ratings of sexual arousal **(A)**, pleasantness **(B)**, and sexual attractiveness **(C)** for female pictures. Error bars represent standard errors of the mean. ^**^p < 0.01.

A significant effect of category on males’ sexual arousal ratings for female pictures was revealed [Welch’s *F*(2, 92.14) = 675.26, *p* < 0.001, est. *ω*^2^ = 0.91; Figure 3A]. The *post-hoc* analysis revealed that males’ sexual arousal ratings for nude female pictures (*M* = 5.39, *SE* = 0.04) than those for semi-nude female pictures (*M* = 4.46, *SE* = 0.05, *p* < 0.001) and dressed ones (*M*= 2.65, *SE* = 0.06, *p* < 0.001; Table 2). In addition, males’ sexual arousal ratings for semi-nude female pictures than those for dressed ones (*p* < 0.001). Unlike females’ ratings for the opposite-sex pictures, these results showed that males rated nude female pictures as more sexual arousal than the other two types of female pictures.

**Table 2:** Males’ sexual arousal, pleasantness, and sexual attractiveness ratings for female pictures.

Ratings	Female pictures		
	Nude	Semi-nude	Dressed
	(Mean ± SE)	(Mean ± SE)	(Mean ± SE)
Sexual arousal	5.39 ± 0.07	4.46 ± 0.07	2.65 ± 0.08
Pleasantness	5.33 ± 0.07	4.38 ± 0.08	2.85 ± 0.08
Sexual attractiveness	5.27 ± 0.07	4.44 ± 0.07	2.85 ± 0.08

#### Pleasantness

Likewise, there was also a significant effect of category on males’ pleasantness ratings for female pictures [Welch’s *F*(2, 89.42) = 473.29, *p* < 0.001, est. *ω*^2^ = 0.99; Figure 3B]. Our *post-hoc* analysis revealed that males’ pleasantness ratings for nude female pictures (*M* = 5.33, *SE* = 0.04) were significantly higher than those for semi-nude female pictures (*M* = 4.38, *SE* = 0.05, *p* < 0.001) and dressed ones (*M* = 2.85, *SE* = 0.07, *p* < 0.001; Table 2). Additionally, males’ pleasantness ratings for semi-nude female pictures were also significantly higher than those for dressed ones (*p* < 0.001). Unlike females’ ratings for the opposite-sex pictures, these findings highlighted that males rated nude female pictures as more pleasant than the other two types of female pictures.

#### Sexual Attractiveness

Finally, a significant effect of category on males’ sexual attractiveness ratings for female pictures was found [Welch’s *F*(2, 93.96) = 506.81, *p* < 0.001, est. *ω*^2^ = 0.89; Figure 3C]. Our *post-hoc* analysis found that males’ sexual attractiveness ratings for nude female pictures (*M* = 5.27, *SE* = 0.04) were significantly higher than those for semi-nude female pictures (*M* = 4.44, *SE* = 0.05, *p* < 0.001) and dressed ones (*M* = 2.66, *SE* = 0.07, *p* < 0.001; Table 2). Furthermore, males’ sexual attractiveness ratings for semi-nude female pictures were higher than dressed ones (*p* < 0.001). Like male sexual arousal and pleasantness ratings of female pictures, males rated nude female pictures as more sexual attractiveness than the other two types of female pictures, which is also distinct from female’s sexual attractiveness ratings for male pictures.

## Discussion

In the current work, we provide a new erotic picture database consisting of neural and erotic photographs depicting East Asian models that are broadly applicable to research in a wide variety of disciplines. These visual erotic stimuli would contribute to the development of research topics aiming to evaluate sex-related functions, especially for future studies involving East Asian samples. Despite this, it is surprising that, to date, almost no empirical studies have attempted to standardize East Asian sexual stimuli. By constructing an erotic picture database along with normative ratings of sexual arousal, sexual attractiveness, and pleasantness, we are hoping to facilitate the processes of erotic stimuli selection and experimental design for future research in human sexuality and non-White samples. Meanwhile, we investigated the sexual response among native Chinese heterosexual college students using our erotic picture dataset. A set of pictures photographing East Asian characters are collected in this study, and it will hopefully facilitate future research on native East Asian participants. In general, the findings support our hypotheses which male and female college students will display significant different preferences for picture categories when viewing both erotic and control ones. Across samples, participants displayed a categorical-specific preference, which supported sex and cultural differences in erotic stimuli processing (Rupp and Wallen, 2008; Ganesan et al., 2020).

### Gender Differences in Response to Opposite-Sex Erotic Stimuli

Our results were consistent with previous studies that males displayed preferences for specific categories of the opposite-sex sexual stimuli. Nude female stimuli received significantly higher ratings than semi-nude female stimuli and control stimuli across all three rating dimensions. In the contrast, females showed a different preference when exposed to the opposite-sex erotic stimuli. Semi-nude male stimuli received significantly higher ratings than nude male ones and control ones across all three rating dimensions. Significant lower ratings on nude male pictures seem to contradict with empirical studies in which western females did not display a preference for the intensity of the opposite-sex erotic stimuli (Chivers et al., 2004). This may presumably be due to the fact that Asians exhibited greater sexual conservatism than Whites (Morton and Gorzalka, 2013; Wu et al., 2016). Given the fact that all the participants were college students recruited from Nanjing University, and the popular believes among Asian parents that sexual behaviors can be a possible distractor of academic performances and thus should be discouraged (Yule et al., 2010), and it is possible that female college students hold more conservative attitude toward sex and extremely explicit erotic stimuli such as naked male pictures.

Since the cultural suppression of sexual expression is possible to impact both heterosexual Asian females and males, a similar pattern of underrating naked female figures should be over served among males. However, it is also intriguing that males in our study reported significantly higher sexual attractiveness and arousal ratings for nude female stimuli. One possible explanation is that heterosexual males tend to watch significantly more pornographies than females. In a study carried among German students, male heterosexual students significantly report higher frequencies of pornography consumption than female students (Böhm et al., 2015). More erotic material consumption indicates that an individual is likely to have an initially positive attitude toward erotic stimuli, such as a naked human body, and mere-exposure effect tends to strengthen the attitudes further. In addition, this difference in self-reported ratings between males and females may be explained by the different mating strategies from the perspective of evolutionary psychology. Favorable attitudes toward sexual cues, such as naked female figures, facilitate male’s reproduction strategy: having more sex partners and offspring. On the contrary, females are supposed to be highly selective about potential sexual partners due to greater initial investment in offspring (Trivers and Campbell, 1972). Factors other than sexuality would be taken into account and favorable attitudes toward sexual cues might not be helpful in this sense. The discrepancy in the ratings between sexes is supposed to reflect the two different mating strategies as a result.

The benefit of using our dataset is that it is possible to select stimuli based on the sex of the participants and the parameter of normative ratings, which would speed up the process of stimuli selection for studies that require stimuli with multiple erotic levels, especially clinical research related to sexual dysfunction such as premature ejaculation and sexual addiction. Our strategy to exclusively collect ratings from heterosexual participants would provide guidance for reliably detecting possible perceptual gaps between college students and patients with sexual disorders from the same sexual orientation. Additionally, the ratings of pleasantness, sexual arousal, and sexual attractiveness can be considered as a guide to calculate sexual reward value for pictures from each category, which would be helpful for studies investigating neuropsychological mechanisms underlying sexual reward in normal and abnormal people (Li et al., 2015; Sescousse et al., 2015).

### Limitations

Despite such encouraging evidence, our study still has some limitations. First, the characters were photographed in a fairly natural manner, thus, as a tradeoff, the pictures were not perfectly standardized. Although facial expression, tones and saturation were controlled carefully by us, the characters’ postures and the background of the images varied across characters. This might slightly affect eye movements if these stimuli are used in an eye-tracking experiment. Second, it is also elusive that to what extent the observed category-specific preferences would be applied from our laboratory settings to real world scenarios, due to the fact that all the stimuli are static pictures. Third, this study included a relative small sample, possibly tempering the strength of our conclusions. Replications with larger samples would be welcome. Fourth, given that aging has a powerful impact on sexual-related behavior (DeLamater and Sill, 2005; Forbes et al., 2017; Johnson et al., 2020) and all participants in this study are young college students, it is thus unclear whether the findings may be generalizable to older people. Future studies need to investigate the role of aging in processing erotic stimuli and its potential interaction with gender and culture. Finally, this study only assessed participants’ ratings for these opposite-sex erotic pictures. However, it is still unknown about how participants rated the same-sex erotic pictures, making it impossible to directly compare these two kinds of ratings in our current study. Therefore, to promote our understanding on this issue, it would be interesting to take it into account in future studies.

## Conclusion

Our study aims to present an East Asian erotic picture dataset that might facilitate future East Asian sexuality research. Meanwhile, when viewing the stimuli from the opposite sex, we found gender differences in ratings of sexual arousal, pleasantness, and sexual attractiveness in response to the opposite-sex erotic stimuli using this dataset. Thus, comparisons of ratings of these three dimensions of erotic pictures in different participant groups will help researchers to choose erotic stimuli dataset for the purpose of various experimental designs.

## Data Availability Statement

The raw data supporting the conclusions of this article will be made available by the authors, without undue reservation.

## Ethics Statement

This study was approved by the Nanjing University Institutional Review Board. The patients/participants provided their written informed consent to participate in this study.

## Author Contributions

YL conceptualized and designed the study and provided critical revisions on it. QC and ZW performed the study and analyzed the data under the supervision of YL. QC drafted the manuscript. ZZ proofread the draft. All authors contributed to the article and approved the submitted version.

### Conflict of Interest

The authors declare that the research was conducted in the absence of any commercial or financial relationships that could be construed as a potential conflict of interest.
